# Negative Effective Mass in Plasmonic Systems

**DOI:** 10.3390/ma13081890

**Published:** 2020-04-17

**Authors:** Edward Bormashenko, Irina Legchenkova

**Affiliations:** Department of Chemical Engineering, Ariel University, Ariel 407000, Israel; ilegchenkova@gmail.com

**Keywords:** metamaterials, negative effective mass, plasma oscillations, low frequency plasmons

## Abstract

We report the negative effective mass (density) metamaterials based on the electro-mechanical coupling exploiting plasma oscillations of a free electron gas. The negative mass appears as a result of the vibration of a metallic particle with a frequency of *ω*, which is close the frequency of the plasma oscillations of the electron gas $m_{2}$ relative to the ionic lattice $m_{1}$. The plasma oscillations are represented with the elastic spring $k_{2}=\omega_{p}^{2}m_{2}$, where $\omega_{p}$ is the plasma frequency. Thus, the metallic particle vibrated with the external frequency *ω* is described by the effective mass $m_{eff}=m_{1}+\frac{m_{2}\omega_{p}^{2}}{\omega_{p}^{2}-\omega^{2}}$, which is negative when the frequency ω approaches $\omega_{p}$ from above. The idea is exemplified with two conducting metals, namely Au and Li.

## 1. Introduction

Metamaterials are recently developed artificial materials demonstrating properties that are not found in naturally occurring materials [1,2]. The domain of metamaterials covers a broad diversity of fields in physics and engineering: electromagnetics, acoustics, mechanics and thermodynamics. In metamaterials, both electric permittivity and magnetic permeability may be negative at certain frequencies [1,2,3]. Moreover, they may be tuned in a broad range of values [4]. The electromagnetic metamaterials are usually synthesized by embedding various constituents/inclusions with novel geometrical shapes and forms in some host media [2]. Various types of electromagnetic composite media, such as double-negative materials, chiral materials and omega media have been studied by various research groups worldwide [1,2,3,4].

A relatively new kind of metamaterials are acoustic metamaterials [5,6,7,8,9,10]. Acoustic metamaterial, in which both the effective density and bulk modulus are simultaneously negative, in the true and strict sense of an effective medium have been reported [5]. Acoustic metamaterials demonstrating the negative Poisson’s ratio have been discussed [10]. Acoustic metamaterials demonstrate a potential to be perfect absorbers of mechanical vibrations [11] and also as materials enabling the focusing of ultrasound [7]. The present paper introduces the negative effective mass metamaterials based on mechano-electromagnetic coupling. The idea of the negative effective mass (density) acoustic metamaterials was demonstrated and discussed in [12,13]. We propose to exploit the plasma oscillations of the electron gas [14] in the development of metamaterials with the negative effective mass (density) [13,15]. The applications of the negative mass (density) materials include: acoustic tunneling through narrow channels, control of the radiation field, perfect transmission through sharp corners and power splitting as discussed in [16]. Elastic wave control and seismic wave protection with acoustic metamaterials possessing the negative mass (density) is considered in [17].

## 2. Results and Discussion

### 2.1. Negative Effective Mass and Plasma Oscillations in Metals

**Figure 1:**
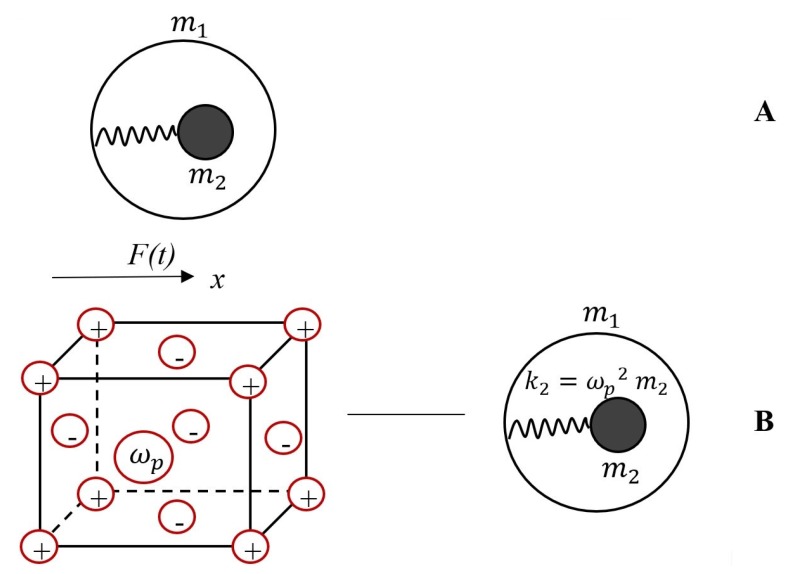
(**A**) Core with mass $m_{2\;}$ is connected internally through the spring with $k_{2}$ to a shell with mass $m_{1}$. The system is subjected to the sinusoidal force $F\left(t\right)=\hat{F}sin\omega t$. (**B**) Free electrons gas $m_{2}$ is embedded into the ionic lattice $m_{1}$; $\omega_{p}$ is the plasma frequency (the left sketch). The equivalent mechanical scheme of the system (right sketch).

The mechanical model, giving rise to the negative effective mass effect, is depicted in Figure 1A. The core with mass $m_{2\;}$ is connected internally through the spring with constant $k_{2}$ to a shell with mass $m_{1}$. The system is subjected to the external sinusoidal force $\left(t\right)=\hat{F}sin\omega t$. If we solve the equations of motion for the masses $m_{1}$ and $m_{2}$ and replace the entire system with a single effective mass $m_{eff}$, we obtain [12,13,15]:

(1) $$m_{eff}=m_{1}+\frac{m_{2}\omega_{0}^{2}}{\omega_{0}^{2}-\omega^{2}}$$

where $\omega_{0}=\sqrt{\frac{k_{2}}{m_{2}}}$. Clearly, when the frequency ω approaches $\omega_{0}$ from above the effective mass $m_{eff}$ will be negative [12,13,15]. Now consider the electro-mechanical analogy of the aforementioned model, giving rise to the negative effective mass. Consider a cubic metal particle, seen as ionic lattice $m_{1}$. containing the Drude-Lorenz free electrons gas possessing a total mass of $m_{2}=m_{e}nV$, where $m_{e}=9.1\times 10^{-31}\;\mathrm{kg}$ is the mass of electron, *n* is the concentration (number density) of the electron gas and *V* is the volume of the particle [14,18,19]. Electron gas is free to oscillate with the plasma frequency $\omega_{p}=\sqrt{\frac{ne^{2}}{m_{e}\varepsilon_{0}}}$ [14,15].

We exposed the entire metal particle to the external sinusoidal force $F\left(t\right)=\hat{F}sin\omega t$. The effective mechanical scheme of the metallic particle is shown in Figure 1B (the right sketch) and it coincides exactly, giving rise to the negative effective mass, supplied in this case by:

(2) $$m_{eff}=m_{1}+\frac{m_{2}\omega_{p}^{2}}{\omega_{p}^{2}-\omega^{2}}$$

where $m_{1}$ is the mass of the ionic lattice, $m_{2\;}$ is the total mass of the electronic gas and $k_{2}=\omega_{p}^{2}m_{2}$; it is seen that it may be negative when the frequency ω approaches $\omega_{p}$ from above. The negativity of the effective mass appears as a result of the attempt to use a single mass $m_{eff}$ to represent a two mass system comprising masses $m_{1},\;m_{2},\;$as noted in [13]. Considering $\frac{m_{2}}{m_{1}}\ll 1$ yields:

(3) $$\frac{m_{eff}}{m_{1}+m_{2}}≅\frac{m_{eff}}{m_{1}}≅1+\frac{m_{2}}{m_{1}}\frac{\omega_{p}^{2}}{\omega_{p}^{2}-\omega^{2}}$$

**Figure 2:**
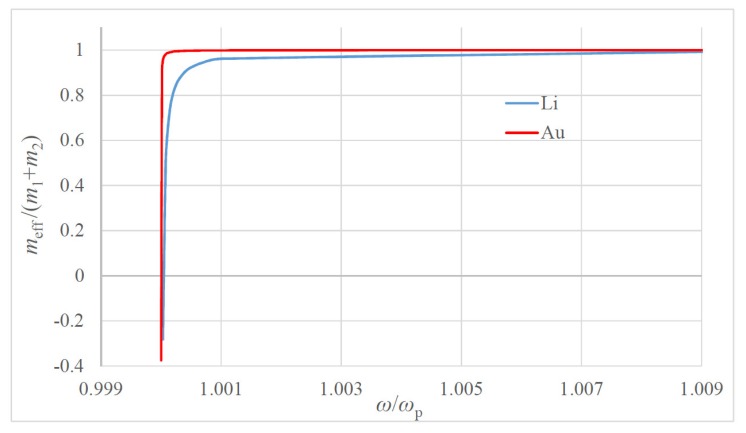
The dependence of the dimensionless mass $m_{eff}/\left(m_{1}+m_{2}\right)$ on the ratio $\omega /\omega_{p}$ is plotted; the red line corresponds to Au; the blue line corresponds to Li.

**Figure 3:**
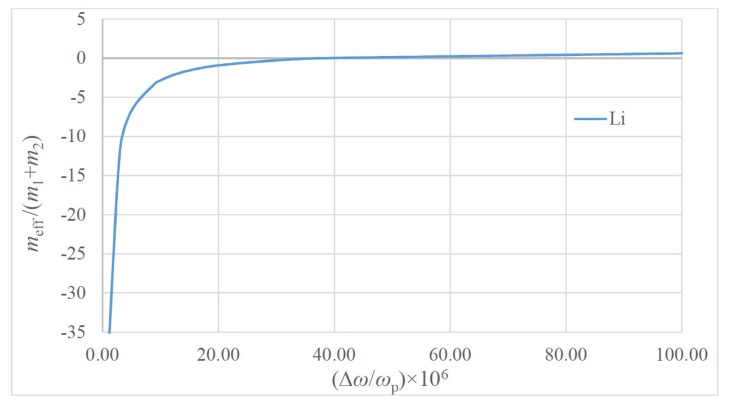
The dependence of the dimensionless effective mass calculated for Li on the $\frac{\omega -\omega_{p}}{\omega_{p}}=\frac{\Delta \omega }{\omega_{p}}$.

**Figure 4:**
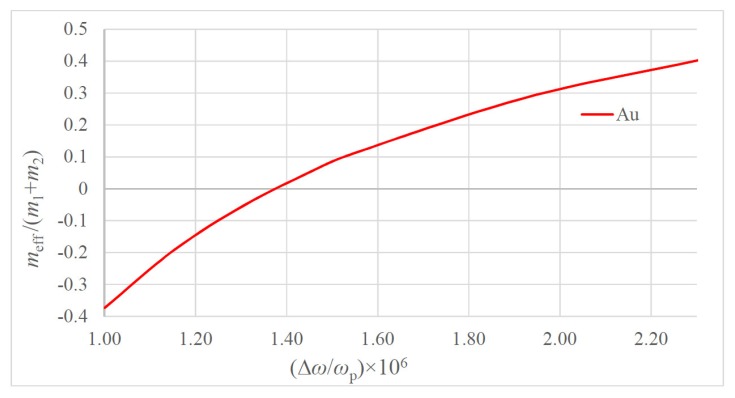
The dependence of the dimensionless effective mass calculated for Au on the $\frac{\omega -\omega_{p}}{\omega_{p}}=\frac{\Delta \omega }{\omega_{p}}$.

It is clear from Equation (3) that the effective dimensionless mass $\frac{m_{eff}}{m_{1}+m_{2}}≅\frac{m_{eff}}{m_{1}}$ depends only on the ratio $\frac{m_{2}}{m_{1}}$; thus, it is independent on the metallic particle size. Thus, for the purposes of calculation, $m_{2}$ is taken as the mass of electron $m_{e}$, and $m_{1}$ is the mass of the atom of metal (see Table 1). The dependence of the dimensionless effective mass $m_{eff}/\left(m_{1}+m_{2}\right)$ on the dimensionless frequency $\omega /\omega_{p}$ for two model metals Li and Au is plotted in Figure 2 (the data relevant to these metals is supplied in Table 1). The macro-scale values of the “plasma spring” constant $k_{2}≅10^{2}\frac{N}{m}$ are noteworthy. The dependencies of the dimensionless effective mass $m_{eff}/\left(m_{1}+m_{2}\right)$ on the dimensionless difference $\frac{\omega -\omega_{p}}{\omega_{p}}=\frac{\Delta \omega }{\omega_{p}}$ calculated for Li and Au are presented in Figure 3 and Figure 4.

### 2.2. Negative Mass and Low Frequency Plasmons in 1D Metallic Meso-Structures

**Figure 5:**
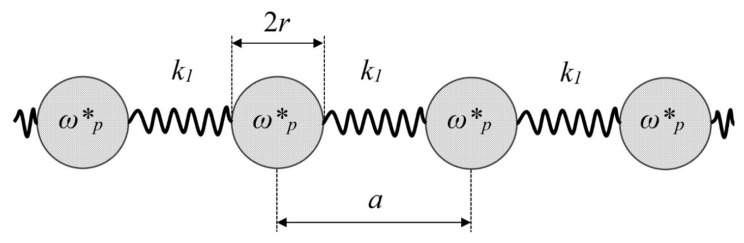
One-dimensional lattice built of metallic wires 2*r* connected with springs $k_{1}$. The separation between wires is *a*.

The plasma oscillations shown in Figure 1 demonstrate the negative mass in the vicinity of the plasma frequency, which is on the order of magnitude of $\omega_{p}≅10^{16}\;\mathrm{Hz}\;$, which is very high. However, this frequency may be decreased very strongly for meso-structures built of thin metallic wires, as demonstrated in [20]. Depression of the plasma frequency into the far infrared and even GHZ band becomes possible due to the mutual inductance that appear in the periodic arrays built of thin metallic wires [20]. We consider the 1D lattice built of the metallic wires with diameter *2r* connected with springs $k_{1}$, as depicted in Figure 5. The effective (pseudo) density of electrons in the metamaterial lattice shown in Figure 5 is given by [20]:

(4) $$\tilde{n}≅\pi n\frac{r^{2}}{a^{2}}$$

where *n* is the concentration of the free electron gas supplied in Table 1 for Li and Au.

The pseudo-mass of electrons in such matrices is given by [18]:

(5) $$\tilde{m}=\frac{\mu_{0}r^{2}e^{2}n}{2}ln\frac{a}{r}$$

where *n* is the concentration of the free electron gas supplied in Table 1. The value expressed by Equation (5) is called in [20] as the “effective mass”; however, in our paper the notion of the “effective mass” is already ascribed to the mass of the vibrated element, given by Equation (1). Thus, we call the value expressed by Equation (5) the “pseudo-mass”, and the effective density of electrons expressed by Equation (4) we label as the “pseudo-density”. Assuming $r=1.0\times 10^{-6}\;m;a=5.0\times 10^{-3}\;m$ we estimate ${\tilde{m}}_{Li}≅6.4\times 10^{-27}\;\mathrm{kg}$; ${\tilde{m}}_{Au}≅8.1\times 10^{-27}\;\mathrm{kg}$. Equations (4) and (5) enable calculation of the effective pseudo-plasma frequencies $\omega_{p}^{*}$ for Au and Li according to Equation (6):

(6) $$\omega_{p}^{*}=\sqrt{\frac{\tilde{n}e^{2}}{\varepsilon_{0}\tilde{m}}}$$

Substituting the aforementioned numerical parameters yields effective plasma frequencies of the lattices built from Au and Li wires $\omega_{p}^{*Au}=4.6\times 10^{10}\;\mathrm{Hz};\;\omega_{p}^{*Li}=5.2\times 10^{10}\;\mathrm{Hz}$, which are much smaller that the aforementioned values of the “true” plasma frequencies.

The spring constants $k_{2}\;$corresponding to aforementioned plasma frequencies are already small and equal *k*_2_(Li) = 2.4 × 10^−9^ N/m, *k*_2_(Au) = 1.9 × 10^−9^ N/m. The optical and acoustical branches of the longitudinal modes propagation in the 1D lattice, depicted in Figure 5, should be elucidated. It should be emphasized that the ensembles of metallic wires, shown schematically in Figure 5, will not demonstrate simultaneously the negative mass (density) and the negative refraction effects [20,21]. This is due to the fact that the negative refraction becomes possible below the plasma frequency $\omega_{p}$ [20,21]; contrastingly, the effect of the negative mass in our model emerges when the frequency ω approaches $\omega_{p}$ from above; thus, the creation of material demonstrating the negative density and dielectric constant simultaneously remains challenging. A more comprehensive approach should consider inevitable losses resulting in the decay of plasmons [22], consequently influencing the effect of the negative mass considerably, as discussed in [23].

## 3. Conclusions

We conclude that exploiting the plasma oscillations of the electron gas relative to the ion lattice gives rise to the negative effective mass phenomenon. The effect takes place when a metallic particle is vibrated with the external frequency ω approaching the plasma frequency $\omega_{p}=\sqrt{\frac{ne^{2}}{m_{e}\varepsilon_{0}\;}}$ from above. In this case, the effective mass of the particle $m_{eff}=m_{1}+\frac{m_{2}\omega_{p}^{2}}{\omega_{p}^{2}-\omega^{2}}$, where $m_{1}\;$is the mass of the ionic lattice, and $m_{2}$ is the mass of the electron gas, becomes negative [12,13,15].

The plasma oscillations may be phenomenologically represented with the ideal spring $k_{2}=\omega_{p}^{2}m_{2}$. Macro-scaled values of $k_{2}≅10^{2}\frac{N}{m}$ for typical metals (namely Li and Au) are noteworthy. The effects, due to the negative effective mass, become possible in the nearest vicinity of the plasma frequencies, inherent for typical metals which are high, namely $\omega_{p}\sim 10^{16}\;\mathrm{Hz}$. The dimensionless effective mass of the particle $\frac{m_{eff}}{m_{1}+m_{2}}≅\frac{m_{eff}}{m_{1}}≅1+\frac{m_{2}}{m_{1}}\frac{\omega_{p}^{2}}{\omega_{p}^{2}-\omega^{2}}$ does not depend on the size of the metallic particle. The plasma frequency may be decreased markedly for the low frequency plasmons predicted for the metallic meso-structures [20], enabling manufacturing metamaterials, which demonstrate the effective negative density. Negative density metamaterials demonstrate the potential of acoustic tunneling through narrow channels, perfect power transmission through sharp corners, elastic power splitting and seismic wave protection [16,17].

## Figures and Tables

**Table 1:** Material constants used in calculations.

Metal	*m*_1_ (kg)	*m*_2_ (kg)	*n* (m^−3^)	*ω_p_* (Hz)	$k_{2}=\omega_{p}^{2}m_{2}\;(N/m)$
Li	1.17 × 10^−26^	9.1 × 10^−31^	4.7 × 10^28^	1.0 × 10^16^	90.0
Au	3.27 × 10^−25^	9.1 × 10^−31^	5.9 × 10^28^	1.3 × 10^16^	152.1
